# Clinical Benefit of Radioiodine Administration in a Rare Case of Iodine Avid Thyroid Carcinoma with No Secretion of Thyroglobulin

**DOI:** 10.1055/s-0043-1777692

**Published:** 2023-12-26

**Authors:** A. Mouaden, H. Guerrouj, I. Ghfir, Ben Rais Aouad N

**Affiliations:** 1 Nuclear Medicine Department, Ibn Sina Teaching Hospital, FMPR, Mohammed V University, Rabat, Morocco

**Keywords:** differentiated thyroid cancer, thyroglobulin, radioiodine treatment

## Abstract

Differentiated thyroid cancer (DTC) is the most common endocrine cancer and its outcome is usually favorable. Its basic treatment is well codified, but its monitoring is much less. The value of thyroglobulin (Tg) is one of the main elements for monitoring DTC, while the use of iodine scintigraphy is becoming less recommended. In this case report, we discuss a clinical situation where a patient presented differentiated thyroid metastatic lesions confirmed by biopsy, uptaking radioactive iodine, with undetectable levels of Tg (in the absence of autoantibodies). We discuss the various hypotheses explaining this clinical situation, the potential advantages of performing periodic iodine scintigraphy in some intermediate and high-risk patients and report the documented clinical benefit of radioiodine therapy.

## Introduction

Differentiated thyroid cancer (DTC) is the most common endocrine cancer and its outcome is often favorable. The basic treatment of classic form of DTC is total thyroidectomy with/ without neck lymph nodes dissection, followed by high-dose radioiodine therapy (I-131 RAI), and thyroid hormone supplementation in a TSH-suppressive or semisuppressive mode, depending on the cancer aggressiveness. It is commonly accepted that the prognosis for DTC is generally favorable if secondary locations are not evident initially or during surveillance. In fact, the 10-year relapse-free survival rate is about 90%, but it drops to 40% when relapse locations occur. 1 The thyroglobulin (Tg) assay is one of the main elements of DTC monitoring. Tg is the precursor protein for thyroid hormone biosynthesis, secreted by thyroid follicular cells and it is an established tumor marker in DTC. Its secretion reflects the thyroid mass and it is stimulated by the thyroid-stimulating hormone (TSH). It is, therefore, expected, after total thyroidectomy and RAI ablation, that the Tg level will be undetectable with the absence of antithyroglobulin antibodies (anti-Tg Abs). Complete remission is defined by a basal Tg inferior to 0.2 ng/mL, or inferior to 1 ng/mL under recombinant human TSH with negative anti-Tg Abs. During follow-up, any increase in Tg level would imply the appearance of recurrence or metastatic locations. Some authors even suggest that serum Tg assay after TSH stimulation is more sensitive than iodine-131 scintigraphy in detecting DTC recurrences and metastases. 2 3

Tg secretion and iodine avidity are supposed to go in the same direction since they both reflect well differentiation. Refractory and undifferentiated thyroid cancers lose both functional abilities. This article discusses an unusual clinical case in our daily practice. This is a patient with DTC who presented iodine avid secondary metastasis, confirmed histologically, with undetectable level of Tg under TSH stimulation (TSH ≥ 30IU/L), and in the absence of anti-Tg Abs. Discussion is raised about biological and molecular hypotheses explaining this discordance. This article also demonstrates the clinical benefit in the use of RAI in the treatment of this kind of DTC presentations.

## Case Report

A 65-year-old female patient underwent a total thyroidectomy surgery 15 years ago for follicular thyroid carcinoma. The patient's clinical and pathological features are summarized in Table 1 .

**Table 1:** Recurrence risk stratification according to clinical and pathological features

Age at diagnosis	65
Histology	Follicular carcinoma Vascular emboli (number of emboli nonreported) Capsular invasion
TNM stage (8th edition, 2017)	pT3a, unifocal N0 M0
AJCC/UICC (8th edition 2017)	Stage II
ATA risk (2015)	Low-to-intermediate risk

Abbreviations: AJCC, American Joint Committee on Cancer; ATA, American Thyroid Association; TNM, tumor node metastases; UICC, Union for International Cancer Control.

**Fig. 1:**
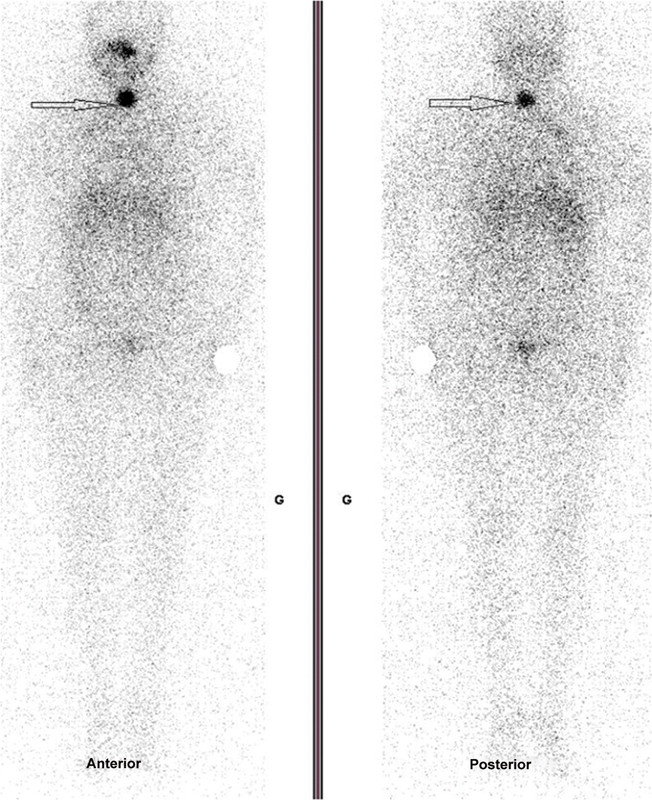
Post-treatment whole body scan showing remnant thyroid tissue ( *arrow* ) with no distant iodine foci.

3.7 GBq of ^131^ I (100 mCi) was administrated to the patient in a radio-protected room after endogenous TSH stimulation, with TSH level controlled at more than 50 mIU/L. The post-treatment scan showed no iodine-fixing locations except for the cervical thyroid area ( Fig. 1 ). Six months later, her diagnostic whole body RAI scan was negative, and Tg was undetectable with negative anti-Tg Abs. The patient was lost to follow-up.

Ten years later, the patient was admitted for a painful mass in the hip and the proximal part of the right thigh. We are informed that she had a total hip prosthesis for a pathological fracture. No data on pathological findings was provided. We requested a computed tomography (CT) assessment and a Tg assay coupled with the anti-Tg Abs assay. CT revealed the presence of a periprosthetic soft tissue mass. Serum Tg measured during suppression with levothyroxine (on T4) and controlled after endogenous stimulation (3 weeks off T4) was negative. The case was discussed in a multidisciplinary meeting and a tumor reduction surgery was decided, for both diagnostic and analgesic purposes.

**Fig. 2:**
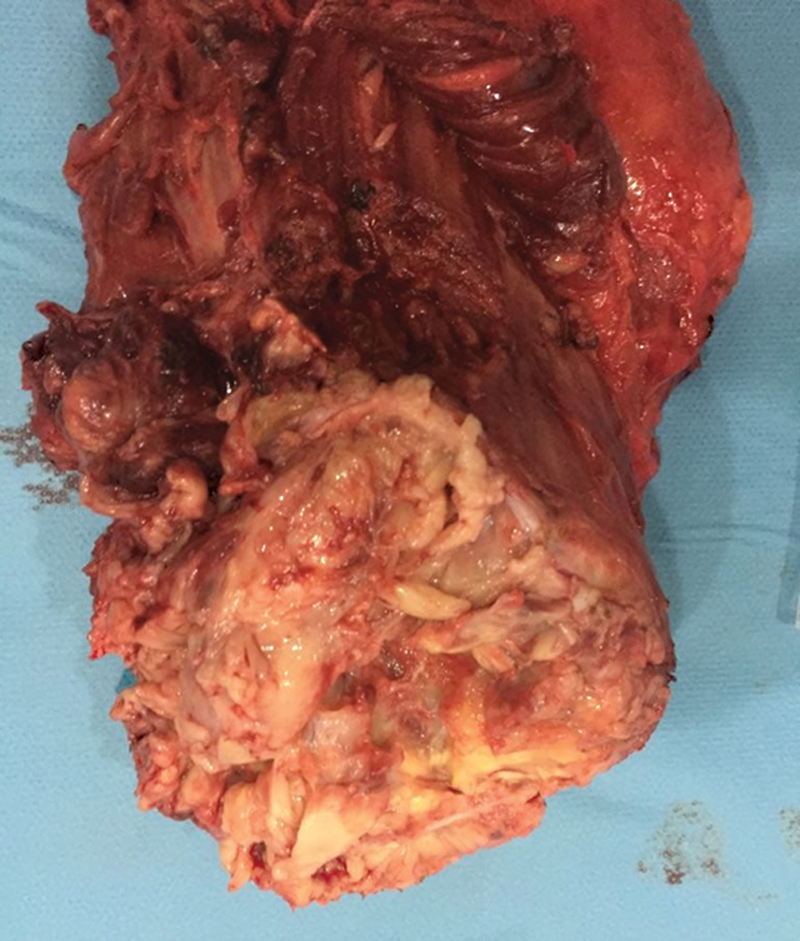
Operative specimen after reduction surgery of the thigh tumor.

**Fig. 3:**
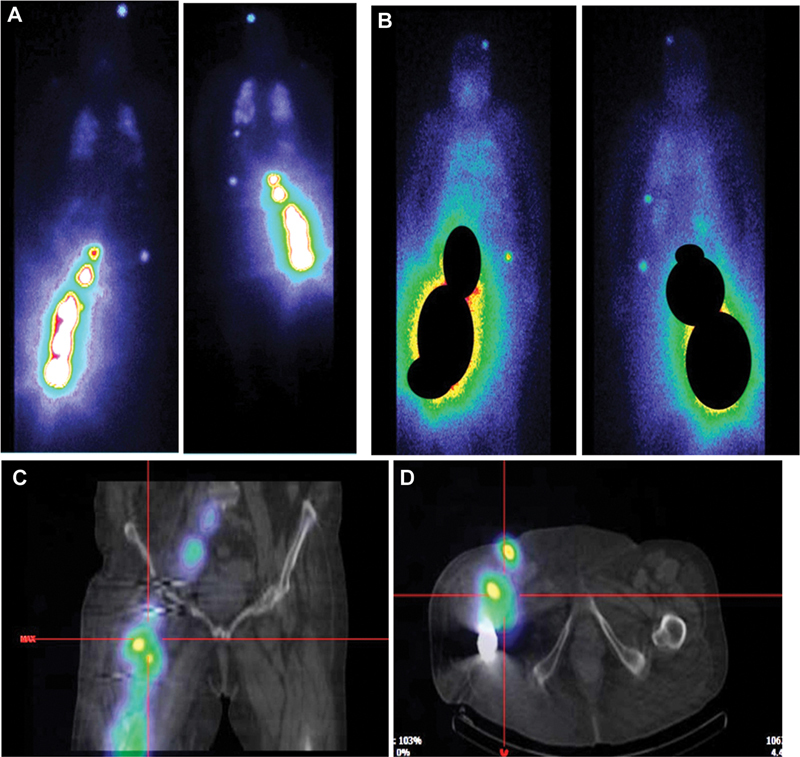
( **A** ) Post-treatment scan after second radioiodine administration. ( **B** ) Post-treatment scan after third radioiodine administration. ( **C** ) Coronal section, single-photon emission computed tomography/computed tomography (SPECT/CT) acquisition after second radioiodine administration. ( **D** ) Axial section, SPECT/CT acquisition after second radioiodine administration.

After surgical excision **(** Fig. 2 **)** immunohistochemical study reported the thyroid differentiated follicular origin of the hip metastasis, expressing TTF1, CK7, CK19, and Tg. A second activity with 5.5GBq (150mCi) of ^131^ I was administered. This activity was well tolerated by the patient and no adverse effects were reported. On post-treatment scanning (whole body and single-photon emission computed tomography/computed tomography acquisitions), several foci and iodine-fixing areas were highlighted in the skeleton, lungs, and soft tissues of the right thigh ( Fig. 3A ).

Despite the low blood Tg level, a third RAI course of 5.5 GBq was decided giving the good iodine impregnation of the metastases and the relative clinical improvement on post-treatment scan ( Fig. 3B ).

**Fig. 4:**
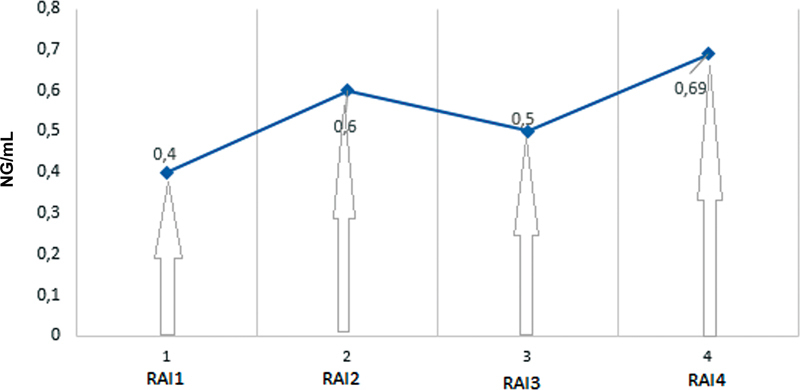
Serum Tg during disease evolution. RAI1: first radioiodine administration, RAI2: second radioiodine administration, RAI3: third radioiodine administration, RAI4; fourth radioiodine administration.

Table 2 and Fig. 4 summarize results and types of assays performed in the dosage of Tg and anti-Tg Abs.

**Table 2:** Summary of assay techniques, FS, and results

	Tg value (ng/mL)	Technique	FS	Anti-Tg Abs value (IU/ml)	Technique	FS
Dosage 1 on T4	0.4	IRMA	0.5–1 ng/mL	6.4	CLIA Architect, Abbot	0.31 IU/mL
Dosage 2 off T4	0.6	Second-generation chemiluminescence Beckman Coulter Access 2	FS < 0.1 ng/mL	< 20	CLIA Architect, Abbot	0.31 IU/mL
Dosage 3 on T4	0.5	Second-generation chemiluminescence Beckman Coulter Access 2	FS < 0.1 ng/mL	< 20	CLIA Architect, Abbot	0.31 IU/mL
Dosage 4 off T4	0.69	Second-generation chemiluminescence Beckman Coulter Access 2	FS < 0.1 ng/mL	17	ALEGRIA	–

Abbreviations: Abs, antibodies; FS, functional sensitivities; CLIA, chemiluminescent immunoassay; IRMA, immunoradiometric assay; Tg, thyroglobulin.

**Fig. 5:**
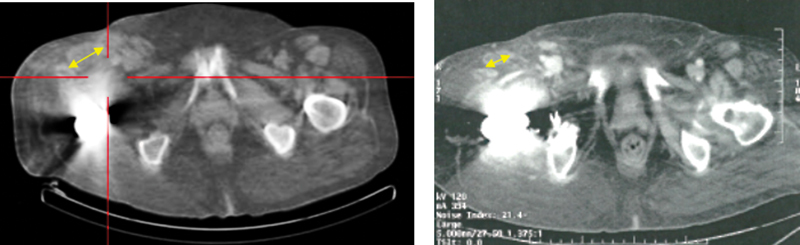
Comparative computed tomography showing the morphological modification in the periprosthetic mass of the right hip and the proximal part of the right thigh ( *yellow double arrows* ).

The patient is currently awaiting the 5th RAI course, with a cumulative activity of 18.5 GBq (500mCi). There was a significant reduction of lung miliary and the thigh's residual soft tissue tumors ( Fig. 5 ).

Despite the persistence of morphological disease, the clinical improvement after 5 years of follow-up is undeniable.

## Discussion

Our patient presented a challenging situation of iodine-avid thyroid cancer metastasis with undetectable Tg. Three main hypotheses may explain this clinical situation: a false positive for iodine, a false negative for Tg, or an early stage of dedifferentiation of the known thyroid neoplasm.

False positive RAI scan may occur since iodine is not specific to thyroid tissue. Many publications have described the pathophysiological mechanisms of RAI uptake by other nonthyroid cells. 4 5 Several explanations are proposed in the literature, including iodine retention in physiological fluids, or the expression of the membrane sodium iodide symporter (NIS), by other healthy nonthyroid cells.

In our case, we concluded to a false-negative Tg for the following reasons:

- The well-differentiated thyroid metastatic origin was histologically confirmed after surgical excision of the right parafemoral soft tissue metastasis.
- The RAI foci mapping on RAI scan suggests a metastatic origin, in particular the pulmonary miliary and bony foci.

The false negative of the Tg is an uncommon phenomenon described in the literature. 6 7 8 In 1990, Brendel et al reported a series of 224 patients with DTC treated by total thyroidectomy and RAI, a percentage of 35% of patients with undetectable serum Tg and positive diagnostic scan. About 8.5% of these patients had metastatic locations other than lymph nodes. 9 Park et al also described a rate of 6.3% of patients with both negative Tg and anti-Tg Abs, with the presence of recurrence on RAI scan (52 patients out of 824). About 86.5% of these patients presented cervical and/or mediastinal lymph node locations. 10 These two studies highlighted an interesting fact: False-negative Tg (in the absence of anti-Tg Abs) is most frequently encountered in small lymph node metastases and without distant localizations, while larger metastatic localizations are associated with higher Tg rates.

Our patient presents very extensive secondary localizations and a massive invasion of the soft tissues, bone, and pulmonary localizations, in the absence of cervical lymph node localization.

Certain phenomena can explain this false-negative Tg:

1) The Tg assay method is not sensitive enough to detect small Tg values. These are situations with low amounts of thyroid tissue especially when TSH is suppressed. Currently, second-generation automated Tg assay methods have a functional sensitivity close to 0.1 ng/mL, detecting DTC relapses earlier. 11
2) The presence of anti-Tg Abs, already mentioned above, leads to an underestimation of the serum Tg value. Radioimmunometric assays are more affected by the presence of autoantibodies. 12 13 The commonly accepted cutoff to certify the negativity of the assay is 100IU/mL. However, some authors consider that a Tg assay is not reliable if the anti-Tg Abs are detected regardless of their serum level. 14
3) The hook effect, associated with immunometric methods, appears when an excessive amount of Tg in the sample exceeds 10 to 10,000 times the upper limit of the reagent's antibodies. A paradoxically weak signal is then obtained. 15
4) Neoplastic tissue produces immunologically inactive Tg: The molecule produced by the tumor is biochemically modified and therefore escapes detection of the Abs used by immunoassays, resulting in falsely low Tg values. This phenomenon has already been described by Brendel et al in cases of poorly DTC metastases. 9 Cells at the onset of “dedifferentiation” could still pick up and concentrate iodine but would be unable to secrete functionally normal Tg. It seems that the loss of iodine concentration function is the last step in tumor dedifferentiation. The last differentiated metastases would be associated with undetectable Tg levels, but would still be able to concentrate iodine. Thus, the function of Tg secretion and that of iodine concentration reflect two different thyroid functions. 16 Hürthle cell carcinoma, for example, is a good secretor of Tg, but has poor iodine binding capacity.

The first two explanations described above do not correspond to the clinical case of our patient since, on the one hand, the tumor mass is “theoretically” responsible for a significant secretion of Tg, exceeding the detection thresholds of the various known assay methods, and on the other hand, the presence of autoantibodies has been ruled out on several occasions and by several assay methods. However, our case could be explained by the last two hypotheses: the hook effect and an early stage of dedifferentiation.

From a therapeutic point of view, our main concern was the relevance of administering high activities of RAI to our patient despite the undetectable level of Tg, and for a purely palliative purpose. We had to do this in the absence of other therapeutic alternatives since the use of thyrosine kinase inhibitors in our country is limited by their cost. A similar reasoning is reported by Zanotti-Fregonara et al who recommend administration of ^131^ I in patients with an undetectable post-surgical Tg. 17 An undetectable Tg level is compatible with RAI therapy when therapeutic benefit is expected and iodine uptake is confirmed by scintigraphy. In the series by Park et al, 47 of 52 patients had one or more high doses of ^131^ I, and the patients showed resolution, improvement, or stability of the lesions, without any case of disease progression. 10

According to the latest recommendations from the American Thyroid Association, iodine scintigraphy is no longer indicated in the follow-up of patients with DTC when the serum Tg is undetectable (in the absence of anti-Tg Ab) and the cervical ultrasound is negative. 18 This is applicable in most low-risk and intermediate-risk patients. For Zerdoud et al, diagnostic ^131^ I scintigraphy may be used in some specific cases such as persistent serum Tg Abs at stable or increasing levels and in high-risk patients. 19 Our patient presented a confusing case in terms of surveillance. The use of diagnostic RAI scan for monitoring would have been beneficial to the early detection of metastasis.

## Conclusion

Through this clinical case, we try to highlight a rare example of limitation in using blood Tg level as the only mean of monitoring DTC, and the potential utility of a periodic RAI scan in intermediate- and high-risk patients. We also advocate the clinical benefit of RAI treatment in a challenging RAI binding metastatic DTC and it utility to delay life-threatening metastasis.
